# The Screening of Therapeutic Peptides for Anti-Inflammation through Phage Display Technology

**DOI:** 10.3390/ijms23158554

**Published:** 2022-08-02

**Authors:** Kangran Zhang, Yezhong Tang, Qin Chen, Yang Liu

**Affiliations:** Chengdu Institute of Biology, Chinese Academy of Sciences, Chengdu 610041, China; zhangkangran18@mails.ucas.ac.cn (K.Z.); tangyz@cib.ac.cn (Y.T.); chenqin@cib.ac.cn (Q.C.)

**Keywords:** phage display, inflammation target, anti-inflammatory peptides, therapeutic peptide, inflammation pathway

## Abstract

For the treatment of inflammatory illnesses such as rheumatoid arthritis and carditis, as well as cancer, several anti-inflammatory medications have been created over the years to lower the concentrations of inflammatory mediators in the body. Peptides are a class of medication with the advantages of weak immunogenicity and strong activity, and the phage display technique is an effective method for screening various therapeutic peptides, with a high affinity and selectivity, including anti-inflammation peptides. It enables the selection of high-affinity target-binding peptides from a complex pool of billions of peptides displayed on phages in a combinatorial library. In this review, we will discuss the regular process of using phage display technology to screen therapeutic peptides, and the peptides screened for anti-inflammation properties in recent years according to the target. We will describe how these peptides were screened and how they worked in vitro and in vivo. We will also discuss the current challenges and future outlook of using phage display to obtain anti-inflammatory therapeutic peptides.

## 1. Introduction

A number of diseases are driven by inflammation, such as rheumatoid arthritis, diabetes, Alzheimer’s disease (AD), cancer, and atherosclerosis, as well as autoimmune, respiratory, and cardiovascular diseases [1,2]. A complex network of numerous mediators, a variety of cells, and several pathways are involved in inflammation. Current therapy for inflammatory diseases is limited to steroidal and non-steroidal medications. Moreover, the anti-inflammatory drugs on the market and used in research usually have significant side effects, particularly when long-term use is involved [3,4].

Finding a safe and effective drug with which to control inflammation represents a significant challenge; therefore, many researchers are committed to developing anti-inflammation drugs. In the past few years, peptides have attracted increasing amounts of attention due to their specific biochemical and therapeutic features, such as diverse bio-functionalities based on their components (amino acids) and high binding affinity with specific targets in a wide range, even though small molecules still dominate the therapeutic industry [5,6]. Peptide discovery optimization has a significant resource advantage over small molecules; the relatively simple and increasing automation of the synthesis required facilitates the success of much smaller teams of medicinal chemists, many-fold smaller than the sizes necessary for a comparable effort in small molecules. A peptide drug is easy to produce and has a lower immunogenicity compared with the antibody. With the improvements made in this technology, the disadvantages of the peptide drug, such as it being membrane-impermeable and biologically unstable, can be solved under certain conditions by direct structural change, enzyme inhibitors, absorption enhancers, carrier systems, and transdermal delivery technologies, promoting peptide drug innovation [7].

Phage display is a powerful tool for developing new peptide drugs, as it can largely maintain the conformations and functions of the expressed protein and peptide simultaneously, thereby maximizing their retention of biological activities with little risk of the recombinant phage infecting the host [8]. The genes expressed on the surface of phages interact directly with various specific targets. For this reason, they are commonly used as a powerful high-throughput screening tool allowing the potential peptide to quickly connect to various specific cellular targets, including membrane receptors and enzymes [9]. To detect ligand–receptor interactions, the displayed phage can be screened against the target proteins immobilized on the enzyme-linked immunosorbent assay (ELISA) plate. In this way, large peptide libraries can be presented on the surface of the phage and panned during repeated cycles, including binding, washing, elution, and amplification. After this, sequencing the genome of the gradually enriched phage provides the display peptide sequence, which can then be used to synthesize the peptide in recombinant or synthetic form. Finally, unique binding agents with a high affinity and specificity for the desired target can be identified [10]. Phage display peptide libraries usually contain up to 10^10^ diverse variants [9]; phages can appear with peptides of a variety of sizes and structures on their surfaces. Natural peptides that are directly separated using traditional separation methods, including high-performance liquid chromatography (HPLC), are usually present in complex mixtures of biological components at relatively low concentrations. Phage display, on the other hand, is more effective and economical in selecting peptide ligands that interact with inflammatory mediators [11,12].

Therefore, it could be effective to use phage display technology to select peptides for anti-inflammation purposes. However, it remains unclear as to whether these peptides are effective candidates for developing medicine to treat disease clinically, which has practical value. In this review, we will summarize the peptides screened for anti-inflammation activity through the phage display technique. Then, we will discuss the current achievements, pros and cons, and prospects relating to this topic.

## 2. How to Use the Phage Display to Screen Peptides

The phage display method was first defined by G. P. Smith et al. in 1985 to express cloned antigens on the viral surface [13]. It is a combinatorial technology that has attracted a great deal of attention regarding its potential in the future of drug discovery. This method is a robust tool in drug discovery, principally for peptide drug identification. It enables researchers to construct libraries and rapidly isolate and identify specific protein interactions of molecular targets [14].

**Figure 1:**
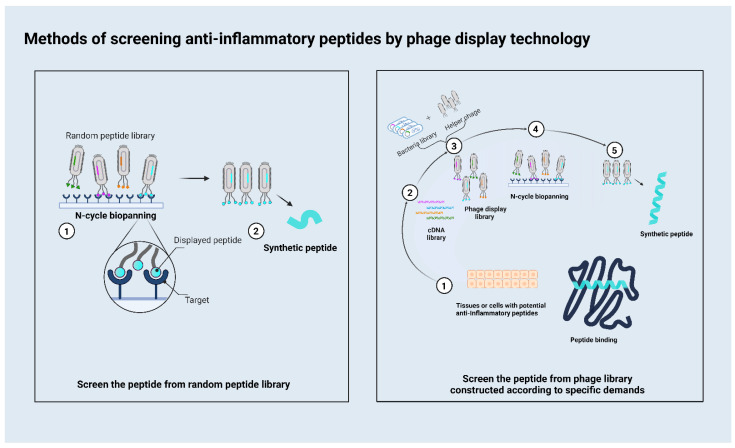
This figure depicts the two most commonly used methods for screening anti-inflammatory therapeutic peptides. The left is the flowchart of screening the peptide from the random peptide library; the right is the flowchart of screening the peptide from the construct library.

Current phage display systems are based on various bacteriophage vectors, including Ml3 phage, T7 phage, λ phage, and T4 phage display systems [15]. The M13 phage display is the most frequently used of these phage systems. There are two main methods used to screen out therapeutic peptides (Figure 1). One employs different targets to obtain a peptide from a random peptide library. In this method, researchers always use different targets associated with inflammation, and the phage display peptide library Ph.D.-7 is the most commonly used library. This method is relatively convenient because it does not require a phage library to be built. Another method involves constructing a phage according to specific demands. For example, some researchers want to obtain functional peptides from a mixture as with a natural product. They would use their mixture to construct a phage that could display these candidate peptides on the surface, then use the target for biopanning to obtain the peptides which have affinity with the target. For researchers aiming to build a new peptide library according to their demands, the T7 system is most likely to be used. As a phage display platform, M13 contains single-stranded DNA, whereas T7 contains double-stranded DNA, which exhibits increased stability and is less prone to mutation during replication. The T7 phage does not depend on a protein secretion pathway in the lytic cycle. Its display system inserts the gene that encodes the specific peptide into its genome so that the target peptide is fused to the C-terminus of the 10B capsid; thus, the target peptide is expressed on the surface of the phage particle, thereby avoiding problems associated with steric hindrance [16]. T7 phage particles exhibit a high stability under extreme conditions, such as high temperatures, and low pH values, which facilitates effective high-throughput affinity elutriation [17].

Although the techniques used to screen the peptides are different, the methods followed to verify their function are quite similar. Firstly, researchers need to verify the affinity between the target and the peptides. Surface plasmon resonance technology (SPR), a major tool used for characterizing and quantifying interactions between biomolecules, is the commonly used and effective way to confirm this affinity. SPR is a technology developed in the 1990s [18], which can monitor the dynamic interaction between ligands and receptors in a fluid environment in real time, so that the affinity constants between ligands and receptors can be calculated [19]. Besides SPR, there are other means to examine the affinity, such as coimmunoprecipitation, but SPR is the most common method used in recent years because it offers exceptional advantages such as being label-free, being able to be used in situ, and providing real-time measurement ability [20].

After confirming the peptides’ affinity, various animal disease models, such as collagen-induced arthritis, lipopolysaccharide (LPS)-induced paw edema, carrageenan-induced paw edema, etc., can be used to verify peptides’ anti-inflammatory activity. In their study, Vogel et al. [21] described many kinds of in vivo and in vitro methods that are used for the pre-clinical assessment of anti-inflammatory drugs. Kalpesh et al. [22] then summarized the advantages and limitations of these animal disease models, so we will not go into detail here.

## 3. The Peptides Obtained according to Different Targets Related to Inflammation

Below, we have listed nearly all the peptides screened for their anti-inflammation properties through phage display in the last 10 years (Table 1). These do not work identically on disease models. The different functions of these peptides depend on the variety of specific targets related to inflammation. According to their different targets, we evaluated some of the peptides screened out for anti-inflammatory properties by describing their screening mechanisms and mechanisms of action in vitro and in vivo.

### 3.1. TNFR1

Primary inflammatory stimuli, including microbial products and cytokines, which mediate inflammation through interaction with the toll-like receptors (TLRs), IL-1 receptor (IL-1R), IL-6 receptor (IL-6R), and the tumor necrosis factor receptor (TNFR) [57], can trigger significant intracellular signaling pathways, including the nuclear factor kappa-B (NF-κB), mitogen-activated protein kinase (MAPK), Janus kinase (JAK) signal transducer, and activator of transcription (STAT) pathways [58,59,60]. To obtain peptides to antagonize the factors in these inflammatory pathways, many researchers have employed inflammatory pathway-related factors as targets to screen the peptides that have an affinity with these factors through phage display. These affinity peptides might have the ability to inhibit downstream signaling pathways and thus could reduce the inflammatory response in vitro and in vivo.

Tumor necrosis factor α (TNF-α) is a multifunctional cytokine [61] which can control the inflammatory process caused by bacterial and viral infections and promote autoimmune diseases as well as cancer [62,63,64]. The biological functions of TNF-α are mediated by two different receptors, TNFR1 and TNFR2, in the cell membrane. To return to homeostasis, the mechanisms that shut down the inflammatory response are of paramount importance [65]. The current research on this topic is focusing on anti-inflammatory drug trends, aiming to identify new small molecules that can directly bind to TNF-α and/or TNFR1 to prevent TNF-α from interacting with TNFR1, thus modulating downstream signaling pathways [66].

However, inhibiting TNF-α occasionally has negative effects, including enabling life-threatening infections, and the reactivation of hepatitis B and tuberculosis [67,68]. In addition, TNF-α blockers cannot show efficacy in diseases where TNF-α acts as a disease-promoting factor, including multiple sclerosis and heart failure. This may reflect the fact that TNF-α blockers prevent not only TNFR1 signal transduction but also the activation of TNFR2 [69,70]. Specifically blocking sTNF/TNFR1 signaling while maintaining the functioning of tmTNF/TNFR2 signaling appears to be adequate to interfere with pathological TNF signaling. The side effects of this class of therapeutics may be less severe than those associated with global TNF blockers that neutralize sTNF and tmTNF and may be effective therapeutic for other diseases, including multiple sclerosis (MS) and neurodegenerative diseases, where it is not recommended to completely inhibit TNF [71].

Therefore, many researchers have used TNFR1 as the target for developing alternative therapeutic interventions rather than TNF-α [72,73]. A peptide Hydrostatin-SN1 (H-SN1) screened from the snake venom of *Hydrophis cyanocinctus* phage libraries not only verified the affinity between SN-1 and TNFR1 but also inhibited the binding between TNF-α with TNFR1 in SPR. Moreover, its anti-inflammatory activities have been verified. H-SN1 suppressed TNFR1-associated signaling pathways by decreasing NF-кB activation and MAPK signaling in HEK293 embryonic kidney and HT29 adenocarcinoma cell lines induced by TNF-α. It has also been found to have an effect in vivo by researchers, using a murine model of acute colitis induced by dextran sodium sulfate, showing that H-SN1 lowered the disease activity index and histological scores in acute colitis and that it could inhibit TNF/TNFR1 downstream targets at both the mRNA and protein levels [74]. The follow-up research on this topic used LPS-induced ALI, LPS-induced bone marrow-derived macrophage (BMDM) cells, and IL-10 knockout mice to test H-SN1’s anti-inflammatory activity; the results suggested that H-SN1 has significant anti-inflammatory effects, both in vitro and in vivo, demonstrating H-SN1 to be a suitable candidate for use in the treatment of TNF-α-associated inflammatory diseases such as inflammatory bowel diseases [24,75]. In addition to H-SN1, A 41-amino acid peptide named DAvp-1 employed TNFR1 as the target, screening it from the T7 phage library of *Deinagkistrodon acutus* venom glands [23]. WH701 was screened from the phage 6-mer peptide library, which is a kind of random library. DAvp-1 and WH701 can both specifically bind to TNFR1 [76,77].

Apart from the peptides screened from the constructed natural product library and random peptide library, a TNFR1-selective antagonistic mutant TNF-α (R1antTNF) was screened from the TNF variant phage library. This research constructed phage libraries which could display the structural TNF variants, where the six amino acid residues (amino acids 29, 31, 32, 145–147, library I; amino acids 84–89, library II) in the predicted receptor binding sites of TNF were replaced with other amino acids. Thus, these phages include many kinds of TNF variant phages. R1antTNF is one TNF variant that can bind with TNFR1 without activating it. This research employed human TNFR1 Fc chimera, which had the same function as TNFR1 but was harder to degrade and easier to separate and purify. According to the results obtained from SPR and x-ray crystallography experiments, although its affinity for the TNFR1 was almost the same as that of the human wild-type TNF, R1antTNF did not activate TNFR1-mediated responses. It also neutralized the TNFR1-mediated bioactivity of wild-type TNF without influencing its TNFR2-mediated bioactivity and could inhibit hepatic injury in an animal model [52]. In later research, the researcher used two independent experimental models induced by carbon tetrachloride or concanavalin A, demonstrating that R1antTNF might be a clinically useful TNF-a antagonist in hepatitis [53]. Referring to the example above, the peptides which blocked TNFR1 had great potential in clinical drug research and development.

### 3.2. CD40

CD40 is an important target belonging to the TNFR family. The CD40/CD40 ligand (CD40L) dyad plays a significant role in several immunogenic and inflammatory processes, including atherosclerosis [78,79,80]. CD40 is expressed by different kinds of cell types relevant to atherosclerosis, including endothelial cells, smooth muscle cells, macrophages, and lymphocytes. CD40 ligation induces a series of inflammatory and apoptotic mediators; hence, CD40 signaling has been associated with the pathophysiology of immunodeficiency, neurodegenerative disorders, collagen-induced arthritis, graft-versus-host disease, atherosclerosis, and cancer. Blocking CD40/CD40L signaling by monoclonal antibodies was shown to be beneficial in the treatment of arthritis and atherosclerosis by disrupting CD40 function, and CD40 has been a key immunotherapeutic target for over 20 years [81].

NP31, which contains a randomized linear 15-mer amino acids peptide sequence, was screened from the human pIF15 phage library using CD40-murine IgG as the target. NP31 inhibits VEGF and IL-6 transcriptional activation and decreases IL-6 production by CD40L-activated endothelial cells. In particular, NP31 was found not only to alter the biodistribution profile of a streptavidin scaffold but also to significantly increase the accumulation of the carrier in aged apolipoprotein e (ApoE) mice with atherosclerosis lesions in a CD40-dependent manner [45]. These instances demonstrate that CD40 could be an available target for screening anti-inflammatory peptides.

### 3.3. IL-17

T helper (Th) cells differ in their cytokine profiles, which identify their subsets. Th1 cells have the characteristics of secret Interferon-gamma (IFN-γ) and TNF-α [82,83]. After the discovery of the Th1 dichotomy, many other Th subsets were discovered, each one having identical functional properties, cytokine profiles, and roles in autoimmune tissue pathology. These Th subsets include Th17 cells, which can produce IL-17 [84].

The IL-17 cytokine family consists of six polypeptides, IL-17A-F, and five receptors, IL-17RA-E1 [85]. This family of cytokines comprises potent inflammatory mediators involved in host defense against extracellular bacteria, fungi, and other eukaryotic pathogens, in which IL-17 cytokines have been implicated in a broad spectrum of inflammatory conditions and autoimmune diseases [86]. IL-17A signals through a specific cell surface receptor complex, which consists of IL-17RA and IL-17RC3, and its downstream signaling leads to an increased production of inflammatory cytokines such as IL-6, IL-8, CCL-20, and chemokine (C-X-C motif) ligand 1(CXCL1) through different kinds of mechanisms, such as the stimulation of transcription and the stabilization of mRNA [87,88,89]. IL-17A and its signaling are significant aspects of host defense against certain fungal and bacterial infections [90,91], and it is an important pathogenic factor in inflammatory and autoimmune diseases. Furthermore, inhibiting IL-17A has preclinical and clinical efficacies in ankylosing spondylitis and rheumatoid arthritis [92,93,94].

HAP is an IL-17A peptide antagonist which was obtained through phage display. Screening followed by saturation mutagenesis optimization and amino acid substitutions produced HAP, which has a high affinity with IL-17A and is able to inhibit the interaction of the cytokine with its receptor, IL-17RA [40]. In the other example, P725 was selected from the phage library of random linear heptapeptides based on their affinity for the target (extracellular domain of IL-7RA, which contains a fibronectin type III repeat-like sequence). P725 had a strong ability to compete with IL-7 for IL-7RA binding sites and can prevent the signal transducer and activator of transcription 5 activations induced by IL-7 in 5-Aza-2′-deoxycytidine (ADC)-stimulated Jurkat cells; thus, it could be a good candidate for blocking applications [41].

### 3.4. IFN-α

IFN-α is a member of the type I IFN family, and IFN-α encompasses 13 partially homologous IFN-α protein subtypes encoded by several genes in humans. All members of this family signal through interferon alpha/beta receptor (IFNAR), a heterodimeric transmembrane receptor comprising IFNAR1 and IFNAR2 subunits, which associate upon ligand binding to activate the protein tyrosine JAK1 and tyrosine kinase 2 (TYK2). This can lead to the phosphorylation of signal transducer and STAT1 and STAT2. Finally, it can associate with IFN regulatory factor 9 (IRF9) and form the IFN-stimulated gene factor 3 (ISGF3). The latter induces the transcription of IFN-stimulated genes (ISGs), with subsequent immunomodulatory effects on both innate and adaptive immune responses [95,96,97]. The IFN pathway, particularly well-documented for IFN-α, has emerged as a major driver of several autoimmune rheumatic diseases encompassing, but not restricted to, systemic lupus erythematosus (SLE) [98], Sjogren’s syndrome (pSS) [99], systemic sclerosis (SSc) [100], and dermatomyositis (DM) [101]. A large amount of evidence supports the important role played by IFN-α in the pathophysiology of several rheumatic autoimmune diseases. Specifically targeting IFN-α or its receptor appears to be a valid approach to ensure its sustained anti-inflammatory efficacy in most patients [102]. To screen a novel IFN-α/β signaling inhibitor to decrease the skin lesions in imiquimod (IMQ) and 12-O- tetradecanoylphorbol-13-acetate (TPA) mice models of psoriasis, one researcher used rabbit polyclonal antibody anti-human IFNα1 as their target to obtain phage peptides (Phpep3D). The derived peptide (Pep3D) reduced skin thickness, redness, and acanthosis despite the presence of the psoriasis inducers IMQ and TPA. Pep3D has also been found to reduce the number of GR1+ infiltrated cells and decrease the production of IL-17A and TNF-α in the psoriatic skin of mice; thus, Pep3D has the potential to be used as a new drug for psoriasis [26], and this demonstrates IFN-α as an effective target.

### 3.5. MMP

In normal cases, TNF-α acts as an immune modulator, and it is a potent inducer of numerous metalloproteinases (MMPs), pro-inflammatory cytokines, adhesive molecules, and chemokines, in which MMPs could increase inflammation [31]. As a kind of zinc-dependent endopeptidase, MMPs break down diverse extracellular matrix compounds [103]. This family of enzymes has multiple common domains in their structure, including a pro-peptide domain, a catalytic domain, and a hemopexin domain in C-terminus, and a fibronectin domain only in MMP-2 and MMP-9 [104]. MMP-2 is an anti-cancer drug target in several aggressive tumors, whereas MMP-9 inhibitors may prove useful in treating cancer in its early stages as well as multiple autoimmune diseases [105]. Abnormal expressions of MMP-2 and MMP-9 are significant factors in some diseases, and so developing an effective inhibitor for the specific and selective inhibition of gelatinases would be helpful. In a study, the researcher chose MMP-2 as a target, to obtain an MMP inhibitor M219hy [32]. In another study, the researcher selected RSH-12 from a library of random 12-mer peptides. RSH-12 could decrease the gelatin degradation by preventing gelatin combinate with MMP-9 and MMP-2. Selective gelatinase inhibitors might prove the usefulness of the new peptide discovered in tumor targeting and anticancer and anti-inflammation therapy [31].

### 3.6. Complement Component 3a (C3a)

The complement system is a significant participant in the innate immune response, where it serves as the initial line of defense against invading pathogens [106]. It might play a crucial role in the immune response and host defense by mediating the activation of immune cells and the eradication of infections [107]. C3a is a thoroughly studied anaphylatoxin that induces proinflammatory reactions together with complement component 5a (C5a). When C3a binds to its receptor, signaling cascades involving C3a are activated, which results in the generation of cytokines and other pro-inflammatory responses. For inflammatory conditions including sepsis and asthma, the inhibition of dysregulated complement activity has been viewed as a possible treatment strategy. This study described the creation of a protein binder that is unique to human C3a (hC3a) and may effectively reduce pro-inflammatory reactions. Six variable sites in the neighboring LRRV2 and LRRV3 modules were subjected to random mutations in order to create a library. Preventing the interaction between hC3a and its receptor, Rb1-H12, which was created through biopanning, had a notable suppressive impact on the proinflammatory response in monocytes. Its specificity to hC3a was shown to be more than ten times greater than that of human C5a [29].

### 3.7. GPR1

GPR1 is a receptor for chemokine-like peptide (chemerin), which is crucial for metabolism and reproduction [108]. Recent studies have shown that GPR1 promotes cancer cell proliferation and invasion in choriocarcinoma cells and gastric cancer cells [109]. The Cancer Genome Atlas (TCGA) has shown a correlation between TNBC and GPR1, especially in TNBC cell lines. TNBC and GPR1 have been shown to be strongly expressed in breast cancer tissue and cell lines, especially in TNBC cell lines [110]. The GPR1-specific peptide LRH7-G5, which competes with chemerin to block chemerin/GPR1 signaling, was screened from the Ph.D.-7 random phage library. The anti-tumor response of this peptide was found to be dose-dependent, inhibiting the proliferation of TNBC cell lines MDA-MB-231 and HCC1937 and suppressing tumor growth, but not T47D cells, through phosphatidylinositol-3-kinase (PI3K)/V-akt murine thymoma viral oncogene homolog (AKT) signaling [30].

### 3.8. CD14

LPS, or endotoxin, is the major structural and functional component of the outer membrane of Gram-negative bacteria [111,112], which has been recognized as the principal component responsible for causing ALI/ acute respiratory distress syndrome (ARDS). These complex macromolecules exhibit a variety of toxic and proinflammatory activities [113]. The proinflammatory role of such low-level LPS relies on the endotoxin-sensitivity enhancing system, LBP/CD14, which is located upstream of the proinflammatory signal path and can pass on and proliferate the LPS proinflammatory signal. Thus, antagonism of the endotoxin-sensitivity enhancing system, LBP/CD14, can efficiently inhibit the proinflammatory role of LPS [114].

In one example, phage display peptide library, phages ELISA and LBP competitive inhibition experiments and DNA screening for testing sequence were jointly adopted, along with the attainment of mimetic peptide sequences (MP12). In both in vivo and in vitro experiments, the biological activity of LPS to cause inflammation was blocked by MP12 and rats suffering from LPS-type ALI were protected by MP12 [115]. In another example, Polypeptide P1, which competes with LBP for CD14 binding, was obtained by screening from a mutant phage display library. It was shown to use error-prone polymerase chain reaction (PCR), induce mutations in the C-terminus of LBP, and attach PCR products to T7 phages. P1 could inhibit LPS-induced TNF-α expression and NF-kB activity in U937 cells and improve arterial oxygen pressure, oxygenation index, and lung pathology scores in rats with LPS-induced acute respiratory distress syndrome (ARDS) [44]. Furthermore, the researcher could use LPS specific antibody as the target to obtain LPS peptide mimics from Ph.D.™-7 Phage Display Peptide Library. This peptide also exhibits certain anti-inflammatory activity [49].

### 3.9. Cell

In addition to proteins, cells can also be used as targets. Using PBMCs as the target could allow us to obtain the heptapeptide HP3 from phage display peptide library Ph.D.-7. HP3 was found to block mononuclear cell adhesion to endothelial cells and inhibit trans-endothelial migration in vitro. The activity of the heptapeptide in a murine model of psoriasis was also assessed, indicating that early administration inhibited the development of psoriatic lesions. Therefore, the results suggested that HP3 may serve as a potential therapeutic target for psoriasis [28].

### 3.10. Others

The above studies uniformly demonstrate that the use of phage display technology to obtain anti-inflammatory peptides is efficient and feasible. The principle of screening is basically to use antagonists of screening targets to antagonize the inflammatory response. Most of these peptides would be used directly after the screening; however, some therapeutic peptides are not directly screened from phages, with researchers instead using peptides screened through phage display and corresponding with other decorations to qualify these peptides. For example, CVX51401 is a Cav modulator that reduces VEGF and immune-mediated inflammation, which fuses RRPPR with a minimal Cav inhibitory domain. In CVX51401, RRPPR was screened from random phage libraries; it was found to be able to internalize efficiently and was demonstrated to be potent in blocking NO release. Caveolin (Cav) regulates various aspects of endothelial cell signaling and cell-permeable peptides (CPPs) fused to domains of Cav can reduce retinal damage and inflammation in vivo. Thus, CVX51401 dose-dependently blocked NO release, VEGF-induced permeability, and retinal damage in a model of uveitis [25]. Taking R1antTNF, which is discussed above, as an example, the molecular stability and bioactivity were improved by converting the homotrimeric R1antTNF into a single-chain derivative (scR1antTNF) through the introduction of short peptide linkers of 5 or 7 residues between the three protomers [116]. The researcher also engineered polyethylene glycol (PEG)-modified R1antTNF (PEG–R1antTNF) to improve stability. As a result of its long plasma half-life, PEG–R1antTNF improved the incidence and clinical score of arthritis. In particular, PEG–R1antTNF showed a greater therapeutic effect than Etanercept in therapeutic protocols. Additionally, it did not reactivate viral infection, unlike Etanercept [54]. This combination means that polypeptides will have more functions and may increase the peptides’ permeability, validity, and stability.

## 4. Conclusions and Future Perspectives

Due to its advantages of a large screening capacity, enabling mass production through fermentation, being high-throughput, and having a straightforward method of execution, phage display has been widely used in bioengineering and biomedicine, especially for diagnostics and therapeutics. With the advent of next-generation sequencing and microfluidic technologies, phage display has become an even more powerful and popular tool for use in drug discovery and development.

However, it also has some limitations. In some constructed libraries, because the peptides displayed on the surface of phages lack modification and the original peptides conformations are different to a certain extent, constructed libraries cannot fully display the original conformations of peptides in vivo. Although the screened peptides have a binding force, they might not play an antagonistic role or even have a therapeutic function. For example, using semaphorin 3F (SEMA3F)/plexin-A2 as the target to screen a peptide with affinity, researchers obtained four peptides AV1, AV2, AcBl3, and AcBl4, which have affinity but which cannot be used in an animal model [117]. We believe there are many peptides, that have not yet been reported, that do not have anti-inflammatory function despite being an inhibitor of the target. Therefore, it is necessary to improve the screening techniques through designs based on experience. Some researchers have combined phage display with other techniques, such as high-throughput sequencing [118], which could help us to better understand and categorize phages after screening.

The therapeutic peptide market is an emerging field that is currently growing, and there are some problems relating to peptide drugs that still need to be solved. For example, the pharmacokinetic properties, the cost of synthetic peptides, and the delivery of peptides to their specific target need to be improved [119,120,121], as these are technical hurdles to the development of more effective peptide-based therapeutics.

Nevertheless, the natural sources or random libraries from which active peptides can be isolated are virtually unlimited. Thus, the appearance of new peptides will not stop soon. According to Craik et al., the market for protein and peptide-based drugs represents about 10% of the total pharmaceutical market, and this proportion is still increasing [120]. Numerous scientific publications demonstrate the intense basic research that is currently taking place in this field, with thousands of peptides being studied as we write, of which 400 to 600 are enrolled in preclinical studies [122]. Although more researchers have used phage displays to screen peptides with anti-inflammatory properties over the past 20 years, the anti-inflammatory peptides developed as drugs are frequently only tested in cells and animals, and clinical trials are required to verify their efficacy. Further research is still needed to improve the effectiveness of screening and the use of peptides as anti-inflammatory drugs.

## Figures and Tables

**Table 1:** The peptides screened for anti-inflammation properties in recent years.

Name/Sequence	Target	Phage Library Type	Properties	References
Davp-1	Tumor necrosis factor receptor 1(TNFR1)	Venom gland T7 phage display library (*Deinagkistrodon acutus*)	Has the affinity with TNFR1.	[23]
Hydrostatin-SN1	TNFR1	Venom gland T7 phage display library (*Hydrophis cyanocinctus*)	Lowers the clinical parameters of acute colitis, including the disease activity index and histologic scores. Reduces inflammation in a mouse model of acute lung injury (ALI) with significant anti-inflammatory effects both in vitro and in vivo.	[24]
CVX51401	Rat heart microvascular endothelial cells (RHMVEC)	Novagen T7 select phage display system	RRPPR is potent in blocking NO release. Fusing RRPPR with a minimal Cav inhibitory domain could dose-dependently block NO release, vascular endothelial growth factor (VEGF)-induced permeability, and retinal damage in a model of uveitis.	[25]
Phpep3D/Pep3D	Rabbit polyclonal antibody anti-human interferon α1 (IFNα1)	Ph.D.™-7 Phage Display Peptide Library	Limits psoriasis-like lesions in mice.	[26]
810A	Thioredoxin-connective tissue growth factor (TrxA-CTGF)	Phage dodecapeptide peptide library	Alleviates fibrosis in the pulmonary index and inhibits inflammation.	[27]
HP3	Peripheral blood mononuclear cells (PBMCs)	Ph.D.™-7 Phage Display Peptide Library	Inhibits the development of psoriatic lesions.	[28]
hC3a-specific protein binder	hC3	Repebody library was constructed by introducing random mutations into six variable sites in nearby two modules, LRRV2 and LRRV3	Suppresses the effect of pro-inflammatory responses in monocytes, by blocking the interaction between hC3a and its receptor.	[29]
LRH7-G5	G protein-coupled receptor 1 (GPR1)	Ph.D.™-7 Phage Display Peptide Library	Suppresses triple-negative breast cancer (TNBC) tumor growth.	[30]
RSH-12	Metalloproteinase 9 (MMP-9)	M13 phage display peptide library (Ph.D.-12)	Decreases the gelatin degradation by specifically preventing gelatin binding to MMP-9 and MMP-2.	[31]
M219hy	MMP-2			[32]
MIT	B cell mimotopes	Random heptamer peptide library	Alleviates allergic responses in a mouse model.	[33]
YSA/SWL	EphA2	M13 phage library displaying random 12-mer peptides	Has the affinity with EphA2.	[34,35]
P-FN12	Anti-H4R antibody	A 12-mer random peptide library	Decreases the production of ovalbumin (OVA)-specific IgE, Th2 immunity, and tissue eosinophilia.	[36]
Anti- inducible T cell costimulatory ligand (ICOSL) variable domain (VNAR)	Antigen	A synthetic VNAR library	Decreases the inflammation of joints, delays overall disease progression, and reduces severity.	[37]
TSL1	gTie2-ectodomain	Ph.D.™-7 Phage Display Peptide Library	Has the affinity with Ang2.	[38]
BKT120Fc and BKT130Fc	Chemokines CCL11, CXCL8, CXCL12, CXCL9, and CCL2	Ph.D-12™ and Ph.D.™-7 Phage Display Peptide Library	Inhibits the ability of inflammatory chemokines to induce the adhesion and migration of immune cells. Inhibits disease progression in a variety of animal models of autoimmunity and inflammation.	[39]
HAP	Interleukin (IL)-17R-Fc	Cyclic and linear peptide libraries	Has the affinity with IL-17R-Fc.	[40]
P725	IL-7Rα	Ph.D.™-7 Phage Display Peptide Library	Competes with IL-7 for IL-7Rα binding sites.	[41]
pm26TGF-β1	Phages that were bound to receptors on the cell surfaces were competitively eluted with 10 ng/mL of recombinant TGF-β1.	Ph.D.™-7 Phage Display Peptide Library	Has direct inhibitory effects on neutrophil migration in a carrageenan-induced peritonitis model.	[42]
ZW1	Aβ42	Ph.D.™-7 Phage Display Peptide Library	Suppresses the inflammatory response by decreasing the release of proinflammatory cytokines, such as tumor necrosis factor α and interleukin 1β, in microglia and reducing microgliosis and astrogliosis in AD transgenic mice.	[43]
P1	A cluster of differentiation 14 (CD14)	LPS-binding protein (LBP) mutants phage peptide library	Reduces the LPS-induced rat lung tissue injury.	[44]
NP31	Human cluster of differentiation 40 (CD40)-murine IgG	pIF15 phage library containing randomized linear 15-mer amino acids peptide sequence	Allows targeted diagnosis of and intervention in inflammatory disorders such as atherosclerosis and autoimmune disease.	[45]
CKGERF and FERKGK	Human chemokine receptor C-C receptor 3 (hCCR3)	6-mer linearpeptidelibrary	Has inhibitory effects on eosinophil chemotaxis in a murine model of mCCL11-induced peritoneal eosinophilia.	[46]
P15-1	Hyaluronan (HA) oligosaccharides	15mer phage display libraries	Attenuates proinflammatory, fibrotic repair by blocking hyaluronan oligosaccharide signaling.	[47]
pep419	Caspase-6	Linear and cyclic peptide phage libraries	Has the affinity with pep419.	[48]
LPS peptide mimics	LPS antibody	Ph.D.™-7 Phage Display Peptide Library	TLR-4 agonist adjuvants.	[49]
PP1	Scavenger receptor A1(SR-AI)	pIF4 phage libraries	SR-AI antagonist.	[50]
CI-S5	PBMCs	Ph.D.™-7 Phage Display Peptide Library	Broad-spectrum antagonist of pro-inflammatory chemokines through enhancing the expression of TTP to reduce chemokine mRNA expression.	[51]
R1antTN	Human TNFR1 Fc chimera	Tumor necrosis factor (TNF) variants in which six amino acid residues	Contains the clinically useful TNF-α antagonist used in hepatitis.	[52,53,54]
SQSHPRH	Inflamed bowel	Ph.D.™-7 Phage Display Peptide Library	Has affinity with inflammatory bowel.	[55]
TM11	Fc-specific goat antihuman IgG	pComb8 phage-displayed peptide library CX15C (in which X is any amino acid and C is a fixed cysteine residue)	New class of small-calcium-dependent P-selectin antagonists based on single-letter amino acid code (EWVDV) core motifs.	[56]

## Data Availability

Not applicable.
